# Elder Mice Exhibit More Severe Degeneration and Milder Regeneration in Temporomandibular Joints Subjected to Bilateral Anterior Crossbite

**DOI:** 10.3389/fphys.2021.750468

**Published:** 2021-12-02

**Authors:** Yuejiao Zhang, Xiaojie Xu, Peng Zhou, Qian Liu, Mian Zhang, Hongxu Yang, Shibin Yu, Jing Zhang, Wanqiu Huo, Yali Zhao, Meiqing Wang

**Affiliations:** ^1^The Key Laboratory of Military Stomatology of State, Clinic of Temporomandibular Joint Disorders and Oral and Maxillofacial Pain, Department of Oral Anatomy and Physiology, School of Stomatology, The National Clinical Research Center for Oral Diseases, The Fourth Military Medical University, Xi’an, China; ^2^The Third Affiliated Hospital of Xinxiang Medical University, Xinxiang, China; ^3^School of Stomatology, Jiamusi University, Jiamusi, China; ^4^First Center of Hepatobiliary Surgery, Fifth Medical Center of the PLA General Hospital, Beijing, China

**Keywords:** aging, occlusion, temporomandibular joint, cartilage, subchondral bone

## Abstract

Temporomandibular joints (TMJs) have a biomechanical relationship with dental occlusion. Aberrant occlusion initiates degenerative remodeling responses in TMJ condyles. Aging is a promoting factor of osteoarthritis (OA) development. The aim of this study was to assess the effect of aging on degenerative remodeling in TMJ condyles in response to occlusal biomechanical stimulation caused by the installation of aberrant prostheses and observe rehabilitation after their removal. The experiments involved 84 female C57BL/6J mice (42 at 6 weeks old and 42 at 28 weeks old). A bilateral anterior crossbite (BAC) model was developed, and the TMJs were sampled at 3, 7, and 11 weeks. BAC was removed at 7 weeks in a subset of mice, which accepted BAC treatment at 6 week of age, and maintained for another 4 weeks after BAC removal. TMJ changes were assessed with micro-CT, histomorphology, immunohistochemistry (IHC), and immunofluorescence staining assays. The results showed that BAC induced typical OA-like TMJ lesions that were more severe in the elder groups as evaluated by the acellular zones, clustered chondrocytes, fissures between cartilage and subchondral bone, reductions in matrix amount and the cartilage thickness as revealed by histomorphological measurements, and subchondral bone loss as detected on micro-CT images. IHC indicated significant increases in cleaved caspase-3-expressing cells and decreases in ki67-positive cells in the BAC groups. There were obvious age-dependent changes in the numbers of superficial zone cells and CD90-expressing cells. Supportively, cleaved caspase-3-expressing cells obviously increased, while ki67-expressing cells significantly decreased with aging. In the elder BAC groups, the superficial zone cells such as CD90-expressing cells were greatly reduced. At 11 weeks, the superficial zone cells were almost non-existent, and there were clear serrated injuries on the cartilage surface. BAC removal attenuated the degenerative changes in the condylar cartilage and subchondral bone. Notably, the rescue effect was more pronounced in the younger animals. Our findings demonstrate the impacts of aging on both TMJ degenerative changes in response to BAC and regenerative changes following BAC removal. The reduced number of chondro-progenitor cells in aged TMJ cartilage provides an explanation for this age-related decline in TMJ rehabilitative behaviors.

## Introduction

Temporomandibular disorders (TMDs) are a widespread problem that affects 5–12% of the population of the United States (Ahmad and Schiffman, 2016). Temporomandibular joint (TMJ) osteoarthritis (OA) is the most severe subtype of TMD (Xu et al., 2020). Animal studies indicated that the amount of extracellular matrix (e.g., type II collagen) is decreased in OA cartilage (Liu et al., 2014; Ye et al., 2018; Tong et al., 2020). Cell death is also promoted as evidenced by increasing levels of cleaved caspase-3 (CCP3) (Zhang et al., 2019). Subchondral bone loss is frequent (Ye et al., 2018; Zhang et al., 2019) and manifests as enhancement of osteoclast-mediated bone resorption that is measurable on micro-CT images (Henriksen et al., 2007; Lu et al., 2015; Zhang et al., 2019). Biomechanical factors and aging are two of the most important initiators of OA (Shen and Darendeliler, 2005; Kuroda et al., 2009; Utreja et al., 2016). Dental occlusion has a close biomechanical relationship with TMJ, and aberrant occlusion can induce TMJ-OA in animal models (Zhang et al., 2018; Yang et al., 2019). Age-associated condylar cartilage degeneration is detectable by histomorphology (Nishijima et al., 2010). It is therefore important to understand the combined effect of dental occlusal biomechanics and aging on TMJ-OA using micro-CT and TMJ histology.

Cells in cartilage are zonally arranged. The superficial zone cartilage harbors cartilage-derived stem/progenitor cells (CSPCs) that express stem cell markers such as CD90 (Dowthwaite et al., 2004; Candela et al., 2014). In the deep zone, cells undergo hypertrophic differentiation, then terminal differentiation, and finally osteogenesis. Degeneration and regeneration are both important events in OA cartilage. In the process of OA disease, there are dominant reductions in the number of chondrocytes and matrix amount. OA also stimulates the intrinsic regeneration capacity mediated by CSPCs (Jiang and Tuan, 2015), although the regeneration efforts are usually unsuccessful (Schminke and Miosge, 2014). Excessive quiescence of the progenitor cells leads to only a few differentiated progenies, whereas excessive proliferation exhausts the stem cell population (Cheung and Rando, 2013; Urbán et al., 2016). It remains unclear how biomechanical factors and aging synergistically affect the intrinsic regeneration capacity of an OA cartilage.

In this study, 6- and 28-week-old mice, representing younger and elder animals, respectively, were subjected to a newly developed dental model termed the bilateral anterior crossbite (BAC) by installing metal tube prostheses onto the mandibular incisors. TMJ responses to BAC were compared between the groups. The rehabilitative behaviors of TMJ cartilage were evaluated following BAC removal. The aim of this investigation was to assess the impact of aging on the degenerative and regenerative remodeling responses in TMJ condyles in response to dental biomechanical stimulation caused by the installation and removal of the aberrant prostheses.

## Materials and Methods

### Groups of Animals and Bilateral Anterior Crossbite Stimulation

**FIGURE 1:**
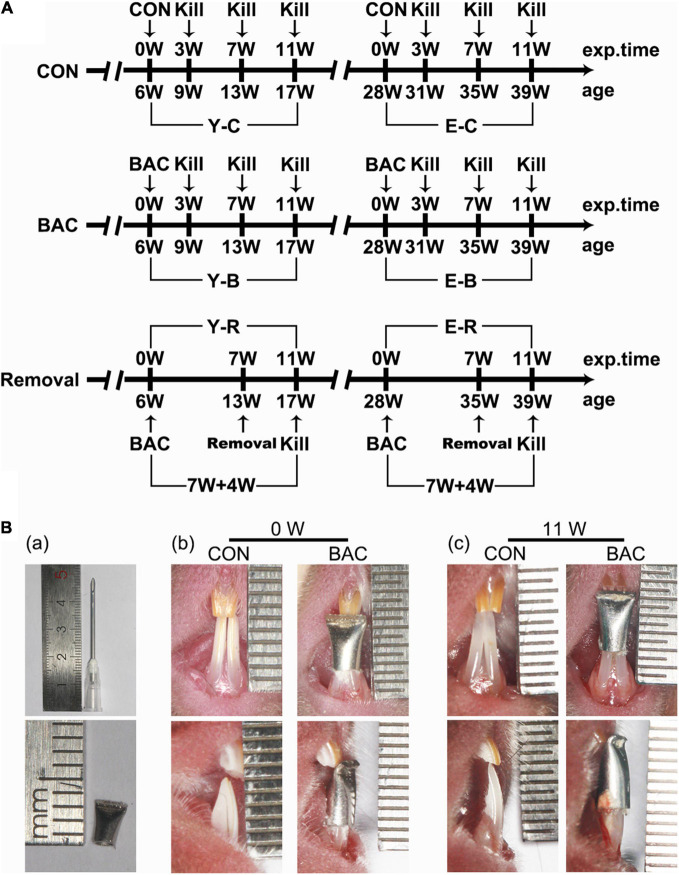
**(A)** The timeline of the entire experiment. **(B)** Representative pinhead and the pinhead produced metal tube (a) used to change the bite of incisors, and frontal views of the incisors in the younger groups with and without the tubed bilateral anterior crossbite (BAC) relationship at 0 week (b) and 11 weeks (c) after modeling. exp. Time, experimental time point; age, the natural age of the mice; CON, control group; BAC, bilateral anterior crossbite group; Removal, BAC removal group. Y-C, younger control group; Y-B, younger BAC group; Y-R, younger removal group; E-C, elder control group; E-B, elder BAC group; E-R, elder removal group. 3, 7, and 11 W: 3-, 7-, and 11-week after BAC installation; 7 + 4 W: 7 weeks of BAC installation followed by the 4-week removal of BAC.

As we did in our previous reports (Zhang et al., 2018), at present, only female animals were used aiming to reduce the impact of sex. Eighty-four C57BL/6J female mice were provided by the Laboratory Animal Center of the Fourth Military Medical University (FMMU). Forty-two of them were younger (6 weeks old, weight 17–18 g) and the other 42 were elder (28 weeks old, weight 22–23 g). The animal study was reviewed and approved by the Administration Committee of Experimental Animals at the FMMU (approval date January 3, 2015, No. 033) and performed according to the Guidelines laid down by the National Institute of Health (NIH) in the United States Guide for the Care and Use of Laboratory Animals. The mice were grouped and sampled as shown in Figure 1A. The younger and elder mice were randomly assigned to three control and four BAC groups (*n* = 6). BAC was created by installing metal tubes onto the mandibular incisors when the animals were 6 weeks old in the younger BAC groups, and were 28 weeks old in the elder BAC groups. Sampling was performed at 3, 7, and 11 weeks. BAC was removed at the seventh week in a subset of mice, which accepted BAC treatment at the age of 6 weeks or 28 weeks, and maintained for another 4 weeks after BAC removal. The cessation of BAC was conducted using the methods as we previously reported (Zhang et al., 2015; Zhou et al., 2020).

### Dental Operations

For the BAC groups, metal tubes that were made of pinheads (1.6 mm, JIANSHI) were bound to cover both the left and right mandibular incisors. The inner diameter of the pinheads was 1.19 mm. The length of the used section of the tubes was 5.00 mm. The tubes were used to cover both the mandibular incisors. And the end of the tubes, about 2.00 mm long, was curved to form a 135° labial inclined occlusal plate to create a crossbite relationship between the bilateral maxillary incisors and the tubed mandibular incisors (Figure 1B-a). The tubes were carefully bonded to the bilateral mandibular incisors. The bite relation of the BAC model at 0 and 11 weeks are presented in Figures 1B-b,c. All the operations were completed within 5 min under anesthetization using 1% pentobarbital (0.4 mL/100 g weight). The prosthesis was checked every other day. No prosthesis fell off during the experimental period. The control mice received the same procedure but without the adhesion of metal tubes. All the animals received the same standardized diet throughout the procedure, and were housed under conditions of 22°C and 30–60% relative humidity with a normal day–night rhythm, consisting of a 12:12 h light–dark cycle.

### Sampling and Tissue Preparation

The mice were sacrificed under deep anesthesia using 1.0% pentobarbital (40 mg/kg). For both the younger and elder mice, the fresh articular surfaces of left-side condyles were observed using stereomicroscope (M250FA, Leica, Hessen, Germany) and then fixed and scanned by micro-CT system (GE explore Locus SP, America) (*n* = 6). After dehydrating in 30% sucrose for 48 h, the condyles without decalcification were prepared for the frozen section and von Kossa (VK) staining (*n* = 6). The right-side TMJ blocks from each subgroup were fixed in 4.0% paraformaldehyde at 4°C overnight. After decalcification in 10% ethylenediaminetetraacetic acid disodium salt solution (EDTA-2Na) for 1 month at 4°C, the TMJ blocks were prepared for paraffin embedding in the sagittal plane, and consecutive central slices were used for histology and immunohistochemistry (IHC) (*n* = 6).

### Micro-Computed Tomography Scanning

**FIGURE 2:**
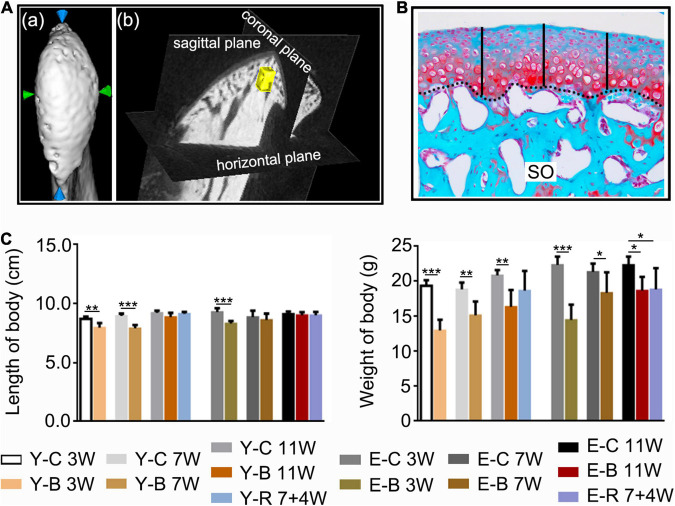
**(A)** The measuring methods for condylar length and width (a), and for the measured regions of interest (ROI, 0.25 mm × 0.25 mm × 0.25 mm) (yellow block) of the subchondral trabecular bone (b) using the software provided by the micro-CT manufacturer. **(B)** The histomorphology measuring method of the cartilage thickness (black-dotted line for the osteochondral interface). Safranin O staining. Middle third of the sagittal sections were selected, three locations at the quintuple points were measured as indicated by the black vertical short lines. **(C)** Comparisons of body length and body weight between the indicated groups at the different time points. Data are presented as mean ± SD. Unpaired *t*-test was used for comparisons between the age-matched control and BAC groups. One-way ANOVA was used for comparisons among the age-matched control, BAC, and BAC removal groups. *n* = 6. *P < 0.05; **P < 0.01 and ***P < 0.001 for the differences between all BAC and removal groups and the age-matched control groups. SO, safranin O; FC, full zone cartilage. For grouping and time points, please refer to Figure 1A.

The left-side condylar samples were fixed overnight and detected under a high-resolution micro-CT system (GE explore Locus SP, America). Samples were scanned coronally at 80 kV and 80 μA with an 8 μm effective pixel size. The sagittal and top images of the condyles were reconstructed using the software (Micview V2.1.2 3D reconstruction processing software). The micro-CT volume was calibrated in the conventional linear scale of CT numbers, Hounsfield units (HU), wherein the values for water and air were 3.2215 and 0.7674, respectively. The width and length of the condylar heads were measured (Figure 2A-a). A cubic section (0.25 mm × 0.25 mm × 0.25 mm) at the center of the condylar subchondral bone (Figure 2A-b) were selected for measurements of BMD, ratio of bone volume to tissue volume (BV/TV), ratio of bone surface area to bone volume (BS/BV), trabecular thickness (Tb.Th), trabecular separation (Tb.Sp), and trabecular number (Tb.N) using the same software.

### Histomorphology and Immunohistochemistry

After micro-CT scanning, the fixed left-side TMJ condyles were prepared for microtomy. Sagittal serial sections were cut at 5 μm using a freezing microtome (HM525NX, Thermo, MA, United States), and then stained with VK as we previously reported (Zhang et al., 2021).

For the right-side condyles, serial sections (5 μm thick) in the sagittal plane of condyles were collected using a rotary microtome (RM2135, Leica, Wetzlar, Germany). Hematoxylin-eosin (HE) staining, Safranin-O/fast green staining (SO), and IHC staining for type II collagen (Col-II) (sc-52658, Santa Cruz, CA, United States) and type X collagen (Col-X) (ab58632, Abcam, Cambridge, United Kingdom) were performed as we previously described (Liu et al., 2019). Moreover, immunofluorescence (IF) staining for analysis of the expression of CCP3 (AF7022, Affinity, United States), Ki67 (ab16667, Abcam, Cambridge, United Kingdom), and CD90 (ab225, Abcam, Cambridge, United Kingdom) was performed as we previously reported (Zhang et al., 2021).

### Histomorphometric Measurements

The HE-, SO-, and IHC-stained sections were observed using a light microscope (DM2500, Leica, Wetzlar, Germany), and imaged using a Leica DFC490 system (Leica, Wetzlar, Germany). Sections with SO staining were used for cartilage thickness and matrix amount measurements as we previously reported (Zhang et al., 2021). The measurements included cartilage thickness (Figure 2B) and size of the SO-positive area. Based on HE- and SO-stained sections, the lesions and recovery of the condyles were assessed using the Osteoarthritis Research Society International (OARSI) grade system by one of the authors (YZ) as we previously described (Duan et al., 2021). The score for the normal mice articular cartilage was 0 and the maximum score for the degenerative articular cartilage was 6.

For the IHC-staining measurements, first of all, a rectangle frame (0.5 mm × 0.3 mm) covering all cartilage layers was applied to the middle third of condyle. Col-II positive area, stained brown, was measured and compared between the groups. The percentage of the Col-X positive cells number to the whole cells number of the farmed cartilage was calculated. For details, please refer to the previous publications (Liu et al., 2014; Zhou et al., 2020; Zhang et al., 2021).

For IF staining, CCP3-, Ki67-, and CD90-positive cells were detected using a laser scanning confocal microscopy (BX-60, Olympus). A square frame (0.3 mm × 0.3 mm) covering all cartilage layers was applied to the middle third of condyle. The number of the total cells, numbers of CCP3-, Ki67-, and CD90-positive cells within the frame were counted. The percentages of CCP3-, Ki67-, and CD90-positive cells to the total cells in the framed cartilage were calculated.

The mean in each sample was used for further statistical analysis.

### Statistical Analysis

All data used were obtained from independent samples or observations. Statistical analysis was performed using Graphpad Prism 7 software (GraphPad company, San Diego, CA, United States). Shapiro–Wilk test was used to assess the normality of data distribution with 95% confidence, and the variance homogeneity was tested using the Levene’s test. To detect the difference between BAC groups and the age-matched control groups, we made the comparisons as follow: all data in BAC groups were compared with the age-matched control groups (*n* = 6) using an unpaired *t*-test; all data in the removal groups were compared with the age-matched BAC groups and control groups using one-way ANOVA (*n* = 6). To detect the changes of CCP3-, Ki67-, and CD90-positive cells with aging in control groups, the percentages of CCP3-, Ki67-, and CD90-positive cells in each time points were compared with that obtained from the younger 3-week control group using one-way ANOVA (*n* = 6). Data acquisition and analysis were performed blindly and expressed as the mean ± SD. Statistical significance was defined as *P* < 0.05.

## Results

### Body Length and Body Weight

Compared with the age-matched control groups, the body length of BAC mice was decreased in younger groups at 3 and 7 weeks and in the elder groups at 3 weeks; body weight was decreased in all the BAC groups and the elder BAC removal group (all *P* < 0.05; Figure 2C).

### Gross Observation and Micro-Computed Tomography Analysis

**FIGURE 3:**
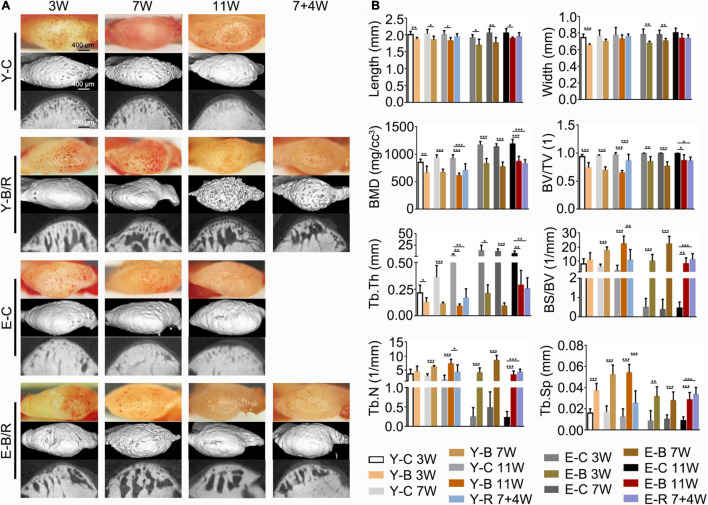
**(A)** Representative gross morphology (bars = 400 μm) and the micro-CT images (bars = 400 μm) of the mandibular condyles. **(B)** Comparisons of the measured parameters as indicated between the BAC groups and the age-matched control groups. Data are presented as mean ± SD. Unpaired *t*-test was used for comparisons between the age-matched control and BAC groups. One-way ANOVA was used for comparisons among the age-matched control, BAC, and BAC removal groups. *n* = 6. *P < 0.05; **P < 0.01 and ***P < 0.001 for the differences between all BAC and removal groups and the age-matched control groups. BMD, bone mineral density; BV/TV, the ratio of bone volume to tissue volume; Tb.Th, trabecular thickness; BS/BV, the ratio of bone surface area to bone volume; Tb.N, trabecular number; Tb.Sp, trabecular separation. For the grouping and time points, please refer to Figure 1A.

Grossly, the condyle surfaces in the control groups were bright and smooth. However, in the BAC groups, the condyle surfaces were congested in the younger groups at 3 and 7 weeks and in the elder groups at 3 weeks. In the 11-week younger BAC group, 7- and 11-week elder BAC groups, and both removal groups, the condyle surfaces appeared matte and rough (Figure 3A).

Compared with the age-matched controls, condylar length in the BAC groups decreased in all younger and elder groups (*P* < 0.05). The condylar width in the BAC groups was decreased in the younger groups at 3 weeks and in the elder groups at 3 and 7 weeks (*P* < 0.05). The subchondral bone was well-arranged in all the control groups. However, in the BAC and removal groups, there was obvious subchondral bone loss and the trabecular space (Figure 3A). Compared with the age-matched control groups, the values of BMD, BV/TV, and Tb.Th were lower in all the BAC groups, while the values of BS/BV, Tb.N (except those in the younger BAC groups at 3 weeks, which showed no significant difference), and Tb.Sp were higher. BS/BV, Tb.N, Tb.Sp, and BV/TV values in the younger removal group recovered compared with the younger 11-week BAC group (all *P* < 0.05), but those in the elder removal group did not. The BS/BV, Tb.N, and Tb.Sp values were higher in the elder removal group and BV/TV was lower than those in the age-matched control group (all *P* < 0.05, Figure 3B). BMD and Tb.Th in both removal groups were lower than those in the age-matched controls (*P* < 0.05).

### Histomorphology

**FIGURE 4:**
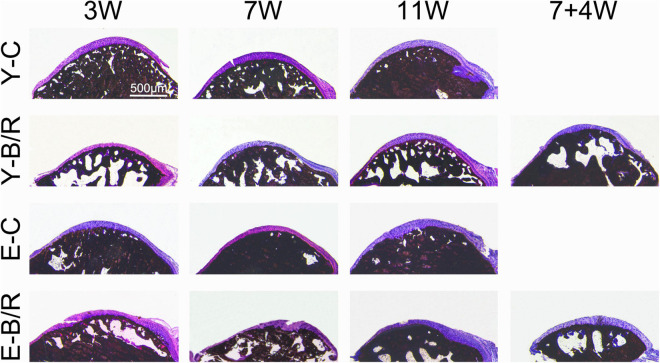
Representative von Kossa (VK) staining. Bars = 500 μm. For grouping and time points, please refer to Figure 1A.

Von Kossa staining revealed that the trabecular bone of the condyles in all the younger control groups was well-arranged, and the bone marrow immediately below the cartilage was shaped in the granular fissures. Conversely, trabecular bone in all the elder control groups was relatively dense with fewer fissures. In all the younger and elder BAC groups, the trabecular bone became sparser, and the bone marrow cavities were significantly enlarged (Figure 4).

**FIGURE 5:**
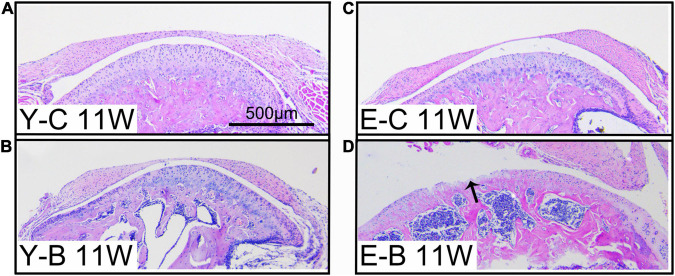
Typical images of younger (Y-) and elder (E-) mice in the control (-C) and BAC (-B) groups at 11 weeks after the BAC operation, labeled as Y-C 11W **(A)**, Y-B 11W **(B)**, E-C 11W **(C)**, and E-B 11W **(D)**, respectively. Bars = 500 μm.

**FIGURE 6:**
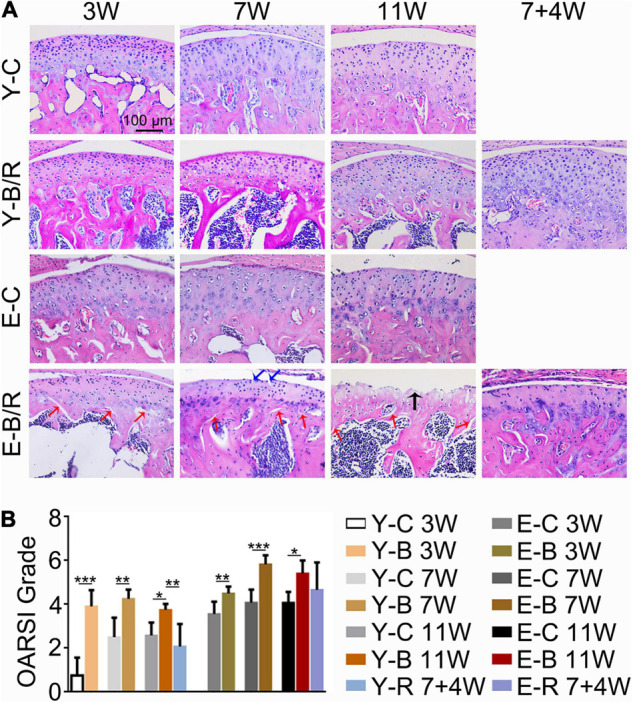
**(A)** Representative hematoxylin and eosin (HE) staining. The black arrow indicates the serrated injury at the surface of the cartilage. The blue arrows indicate the fissures at the surface of cartilage. The red arrows indicate the fissures between cartilage and subchondral bone. Cell-free areas are obvious in the E-B/R groups. Bars = 100 μm. **(B)** Comparison of the OARSI grade between the groups. Data are presented as mean ± SD. Unpaired *t*-test was used for comparisons between the age-matched control and BAC groups. One-way ANOVA was used for comparisons among the age-matched control, BAC, and BAC removal groups. *n* = 6. *P < 0.05; **P < 0.01 and ***P < 0.001 for the differences between all BAC and removal groups and the age-matched control groups. For grouping and time points, please refer to Figure 1A.

**FIGURE 7:**
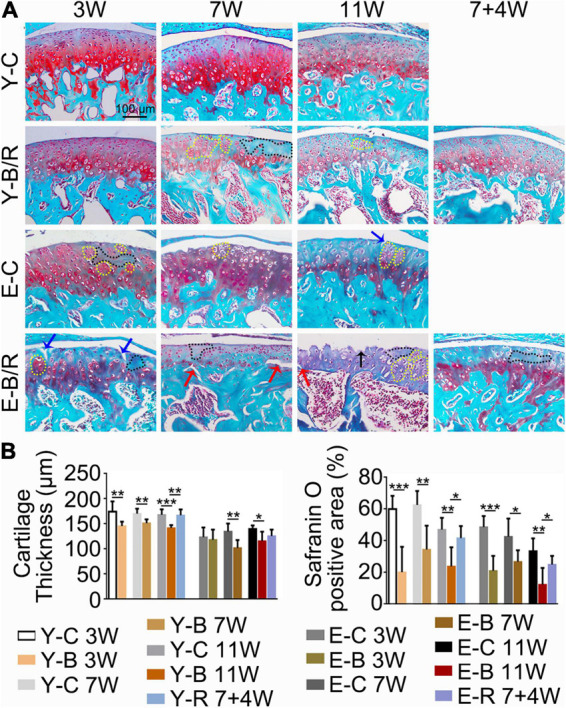
**(A)** Representative Safranin O (SO) staining. The black arrow indicates the serrated injury at the surface of the cartilage. The blue arrows indicate the fissures at the surface of the cartilage. The red arrows indicate the fissures between cartilage and subchondral bone. Cells in the black-dotted areas are lacking. Cells in the yellow dotted areas are clustered. Bars = 100 μm. **(B)** Comparison of the cartilage thickness and the size of the SO-positive area between the groups. Data are presented as mean ± SD. Unpaired *t*-test was used for comparisons between the age-matched control and BAC groups. One-way ANOVA was used for comparisons among the age-matched control, BAC, and BAC removal groups. *n* = 6. *P < 0.05; **P < 0.01 and ***P < 0.001 for the differences between all BAC and removal groups and the age-matched control groups. For grouping and time points, please refer to Figure 1A.

As revealed by HE and SO staining (Figures 5–7), the cartilage surfaces were intact and continuous in the younger control groups. The chondrocytes were arranged in an appropriate orientation with few signs of cluster proliferation. In the elder control groups, however, cells were unevenly and hypertrophically differentiated. Numerous hypertrophic chondrocytes were located in the middle and even superficial regions of the cartilage, with frequent cell clusters. The cartilage was not evenly calcified in thickness, manifesting as cartilage protruding in some sites of the subchondral bone (Figures 5, 6). The cartilage surfaces were generally intact and continuous in the younger BAC groups, although cell-free areas and clustered proliferation were observed. There was a significant reduction in the matrix amount and cartilage thickness in all the younger BAC groups compared with age-matched controls (Figure 7). In the elder BAC group, cartilage degeneration was more significant as evidence by reductions in cell number, matrix amount, and cartilage thickness. Cell-free areas were also frequently noticed. In the 11-week BAC group, there were cartilage surface erosions that appeared as serrated injuries, so that the cartilage surface was no longer continuous (Figures 5–7). Horizontal fissures between the cartilage and subchondral bone were also observed (Figures 6, 7). The OARSI grade was much higher in all the BAC groups compared with the age-matched control groups (Figure 6).

Compared with the age-matched BAC groups, cartilage health was much improved in the younger removal group as evaluated by the cartilage thickness, matrix amount, and OARSI grade (*P* < 0.05). The elder removal group also showed improvements in histomorphology and the matrix amount; however, the cartilage thickness and OARSI grade showed no significant changes (*P* > 0.05; Figures 6B, 7B). There were no cartilage surface ulcers observed in the elder removal group, while the tidemark at the osteochondral interface was significant as revealed by the HE staining (Figures 6, 7).

### Immunohistochemistry and Immunofluorescence Staining

**FIGURE 8:**
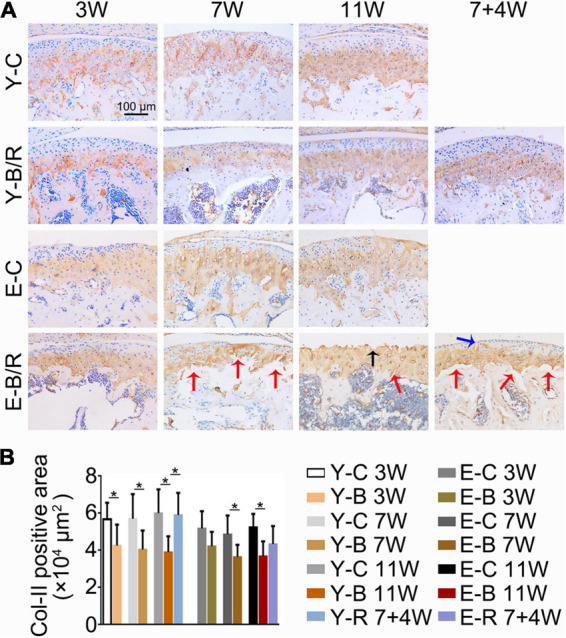
**(A)** Representative immunohistochemical staining of type II collagen (Col-II). The black arrow indicates the serrated injury at the surface of the cartilage. The blue arrow indicates the fissure at the surface of cartilage. The red arrows indicate the fissures between the cartilage and subchondral bone. Bars = 100 μm. **(B)** Comparisons of Col-II positive area between the groups. Data are presented as mean ± SD. Unpaired *t*-test was used for comparisons between the age-matched control and BAC groups. One-way ANOVA was used for comparisons among the age-matched control, BAC, and BAC removal groups. *n* = 6. *P < 0.05 for the differences between all BAC and removal groups and the age-matched control groups. For grouping and time points, please refer to Figure 1A.

**FIGURE 9:**
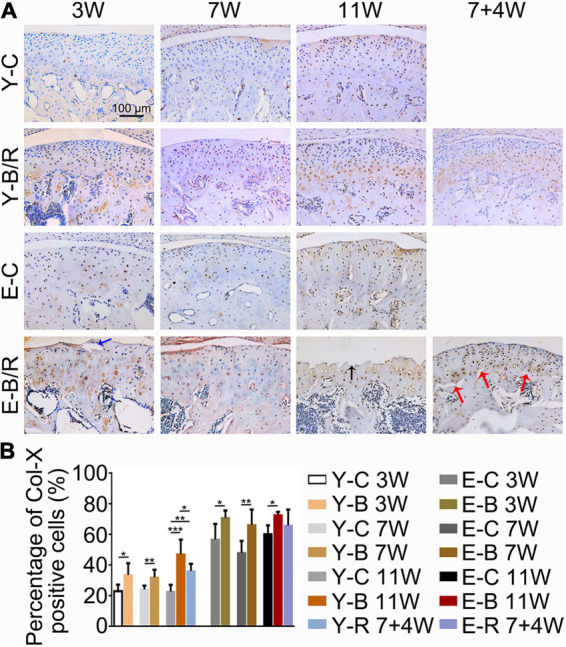
**(A)** Representative immunohistochemical staining of type X collagen (Col-X). The black arrow indicates the serrated injury at the surface of the cartilage. The blue arrow indicates the fissure at the surface of cartilage. The red arrows indicate the fissures between cartilage and subchondral bone. Bars = 100 μm. **(B)** Comparisons of the percentages of Col-X positive cells in the cartilage between the groups. Data are presented as mean ± SD. Unpaired *t*-test was used for comparisons between the age-matched control and BAC groups. One-way ANOVA was used for comparisons among the age-matched control, BAC, and BAC removal groups. *n* = 6. *P < 0.05; **P < 0.01 and ***P < 0.001 for the differences between all BAC and removal groups and the age-matched control groups. For grouping and time points, please refer to Figure 1A.

The Col-II positive area was smaller in all BAC groups at 7 and 11 weeks compared with the age-matched control groups. In the younger removal group, the Col-II positive area increased to the level of the younger 11-week BAC group, but there was no improvement in the elder removal group (Figure 8). Similarly, the percentages of Col-X positive cells to total cells were increased in all BAC groups compared with the age-matched control groups. The percentage of Col-X positive cells was greatly recovered in the younger removal group (*P* < 0.05) but not the elder removal group (Figure 9).

**FIGURE 10:**
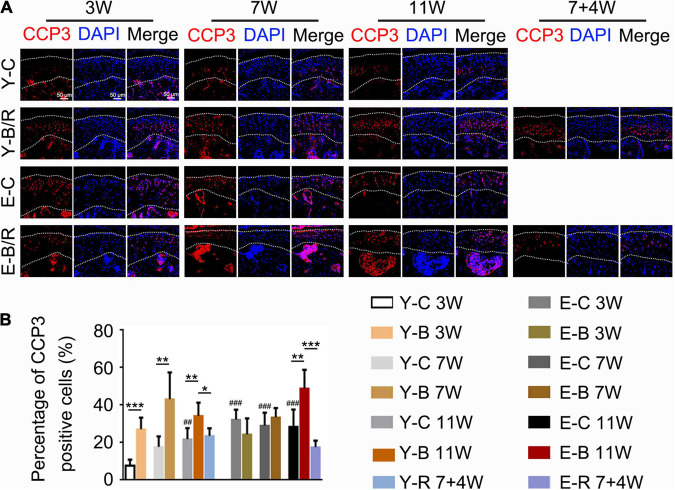
**(A)** Representative cleaved Caspase-3 (CCP3) immunofluorescence staining. Bars = 50 μm. **(B)** Comparisons of the percentages of CCP3-positive cells in the cartilage between the groups. Data are presented as mean ± SD. Unpaired *t*-test was used for comparisons between the age-matched control and BAC groups. One-way ANOVA was used for comparisons among the age-matched control, BAC, and BAC removal groups, and the comparisons among the six time points in control groups. *n* = 6. *P < 0.05, **P < 0.01 and ***P < 0.001 for the differences between all BAC and removal groups and the age-matched control groups. ^##^*P* < 0.01, ^###^*P* < 0.001 for the differences between each time points control groups and the younger 3-week control group. For grouping and time points, please refer to Figure 1A.

Cleaved caspase-3 positive cells were predominantly distributed in the deep cartilage region in the younger control mice, but they were observed throughout the cartilage in the younger BAC groups and both the BAC and control elder groups (Figure 10A). The percentages of CCP3 positive cells to total cells were identical between the 3- and 7-week younger control groups, but were higher in 11-week younger control group and in the elder control groups at all the three time points taking that of the 3-week younger control group as the reference (*P* < 0.05). The percentages of CCP3-positive cells to total cells in all the younger BAC groups and in the elder 11-week BAC group were much higher than the age-matched control groups, but they normalized in both removal groups (all *P* < 0.05; Figure 10B).

**FIGURE 11:**
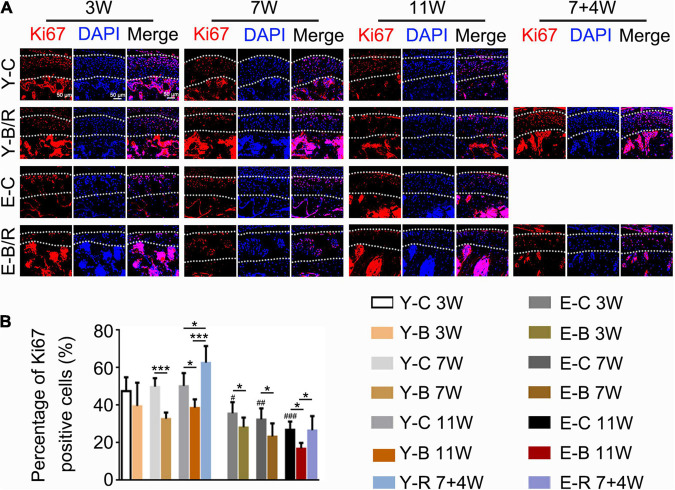
**(A)** Representative Ki67 immunofluorescence staining. Bars = 50 μm. **(B)** Comparisons of the percentage of Ki67-positive cells in the cartilage between the groups. Data are presented as mean ± SD. Unpaired *t*-test was used for comparisons between the age-matched control and BAC groups. One-way ANOVA was used for comparisons among the age-matched control, BAC, and BAC removal groups, and the comparisons among the six time points in control groups. *n* = 6. *P < 0.05, ***P < 0.001 for the differences between all BAC and removal groups and the age-matched control groups. ^#^*P* < 0.05, ^##^*P* < 0.01, ^###^*P* < 0.001 for the differences between each time points control groups and the younger 3-week control group. For the grouping and time points, please refer to Figure 1A.

The Ki67-positive cells were predominantly distributed in the superficial zone cartilage in all groups (Figure 11A). The percentages of Ki67-positive cells to total cells were identical between the three younger control groups, but were lower in all elder control groups taking that of the younger 3-week control group as the reference (*P* < 0.05). The percentages of Ki67-positive cells to the total cells in the 7- and 11-week younger BAC groups and in all the elder BAC groups were much lower than the age-matched controls, while those in the two removal groups were both recovered (all, *P* < 0.05; Figure 11B).

**FIGURE 12:**
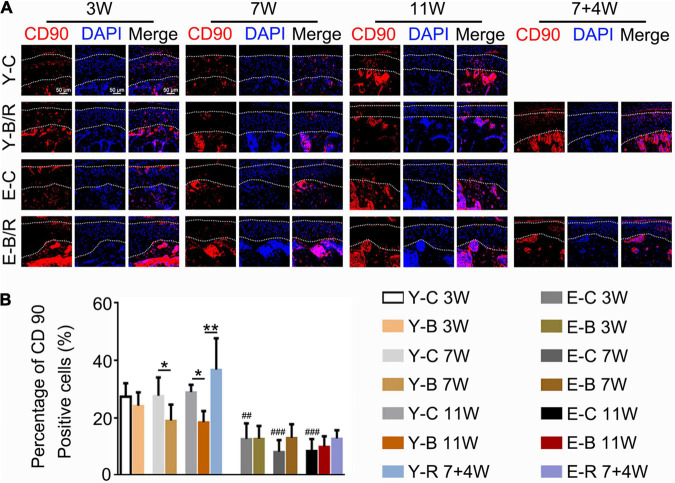
**(A)** Representative CD90 immunofluorescence staining. Bars = 50 μm. **(B)** Comparisons of the percentage of CD90-positive cells in the cartilage between the groups. Data are presented as mean ± SD. Unpaired *t*-test was used for comparisons between the age-matched control and BAC groups. One-way ANOVA was used for comparisons among the age-matched control, BAC, and BAC removal groups, and the comparisons among the six time points in control groups. *n* = 6. *P < 0.05, **P < 0.01 for the differences between all BAC and removal groups and the age-matched control groups. ^##^*P* < 0.01, ^###^*P* < 0.001 for the differences between each time points control groups and the younger 3-week control group. For grouping and time points, please refer to Figure 1A.

CD90-positive cells were predominantly distributed in the superficial cartilage layers in all the groups (Figure 12A). Compared with the younger 3-week control group, the percentages of CD90-positive cells to total cells were identical between the three younger control groups but lower in all three elder control groups (*P* < 0.05). These values in the younger BAC groups at 7 and 11 weeks were lower than those in the age-matched control groups, but they completely recovered in the younger removal group (all *P* < 0.05). These patterns of CD90-positive cell reduction and recovery were not observed in the elder removal group (*P* > 0.05; Figure 12B).

## Discussion

Presently, the TMJ responses to BAC stimulation were compared in the younger and elder animals. The results demonstrated that BAC-induced degenerative responses in TMJs, showing as the reduction in cartilage thickness and cell number, formation of the chondrocytes clusters and appearance of the fissures in cartilage as we indicated in Figure 7. The degenerative changes were more obvious in the elder BAC group. Surface ulcers were only observed on the articular condylar cartilage in the 11-week BAC elder group. To the best of our knowledge, this is the first evidence that dental occlusion induced ulcers on the articular surface of TMJ condyles. Furthermore, the regeneration response was milder in the elders than that in the younger group. The Ki67 expression level in the younger BAC mice was not reduced until 7 weeks while that in the elder group decreased significantly from 3 weeks. Removal of the metal tubes attenuated the degenerative lesions, and the rescue effect was more obvious in the youngers than in the elders. Our findings indicate that aging significantly impacts the degenerative and rehabilitative responses in the TMJ to BAC installation and removal.

In rodents, the mandibular incisors function in a gliding movement under the guidance of the lingual surface of the maxillary incisors. During the incising process, the mandible is directed to move from labial to lingual with more or less lateral movements. The normal overlap relationship of the bite facilitates a sagittal gliding movement, as well as the minor lateral chewing movement of the mandible. The normally labial overjet features of the maxillary incisors prevent the incisors from interferential contacts during the lateral deviations of the mandible. However, under the condition of BAC, the tubed mandibular incisors with the labially inclined occlusal plate not only changed the incising movement to lingual-to-labial direction, totally opposite to the normal, but also may interrupt the lateral rotational movements of the mandible, even though the lateral rotation extension is considered small in mice. The stress over condyles will then be changed in response to BAC, although the types of mechanical loading are unknown because of the limited loading measuring techniques. The net outcome is that condyles experienced excessive or aberrant loading when the mandible is moved during the chewing process. The altered condylar loading ultimately causes TMJ degeneration. The OARSI grades and the CCP3-expression level in cartilage were not higher in the younger 11-week BAC group compared with the younger 7-week BAC group, as shown in Figure 6. We owe this limited degradation in the BAC model to the untubed maxillary incisors. The natural maxillary incisors in the BAC model were promoted to be worn by the tubed mandibular incisors. Such progressive tooth wear should slightly neutralize the negative impact of BAC on TMJs, thus, contributing to higher rehabilitative responses or no worsening of degeneration in the younger 11-week BAC group compared with the younger 7-week BAC group.

The superficial zone cartilage harbors chondro-progenitors. It was reported that these cells actively proliferate and were functionally promoted to differentiate into mature chondrocytes, and then into osteoblasts and osteocytes, similar to the process of endochondral osteogenesis (Hirata et al., 2009; Liu et al., 2014; Yang et al., 2014; Jing et al., 2015). We previously reported a bilateral anterior elevation (BAE) model that was created by installing metal tubes onto the four incisors of mice, giving the tubed incisors an edge-to-edge bite relation. BAE induced a proliferative response in the mandibular condylar cartilage, characterized by increases in the cell number, matrix amount, and cartilage thickness (Liu et al., 2019). This proliferative behavior makes BAE a useful tool to promote rehabilitation in the degraded cartilage (Zhou et al., 2020). In cartilage under osteoarthritic changes, chondro-progenitors are stimulated to proliferate or differentiate (Jiang et al., 2016). However, in the present BAC model, the percentage of the CD90-expressing cells was decreased compared with the age-matched controls. Severe degeneration with milder rehabilitation in the BAC-treated TMJ cartilage agreed well to this reduction of the CD90-expressing chondro-progenitors. Furthermore, our results showed that the percentage of the chondro-progenitors was age dependent. The percentages of CD90-expressing cells to total cells in cartilage were ∼30% (27.39–28.95%) in the younger control groups, but ∼10% (8.23–12.74%) in the elder groups. As a consequence, the percentage of CD90-expressing cells in TMJ cartilage was significantly increased after BAC removal in the younger group, even though their numbers had indeed been reduced by BAC. In the elder groups, however, BAC and its subsequent removal had the least impact on the percentage of CD90-expressing cells. Actually, there were fewer superficial zone cells in the elder removal group. The shortage of chondro-progenitors in the elder groups explains the more severe degeneration induced by BAC and the decline of the rehabilitative responses following the BAC removal.

Mouse and its masticatory system are obviously not identical to humans. The present BAC prosthesis in a mouse cannot be equal to the anterior crossbite in humans, even though the histological structure of the articular cartilage of mice is overall similar to that of humans (Porto et al., 2010). Animal studies are usually used for the TMJ investigation because it is impossible to get sufficient human samples. The data from animal TMJs are helpful in the exploration of the histological and molecular mechanisms of diseases. As for our data, an age-related reduction of the chondro-progenitor cells in TMJ cartilage provides an explanation for the age-related decline in biomechanically stimulated TMJ cartilage, although the answers cannot be finalized unless the gain-and-lost strategy was adopted, such as using the technique that target interferes the chondro-progenitors.

## Conclusion

Compared with the younger animals, there were more severe degenerative responses to aberrant BAC dental biomechanical stimulation and much weaker regenerative responses to BAC removal in the elder mice. An age-related reduction of the chondro-progenitor cells in TMJ cartilage provides an explanation for this age-related decline in biomechanically stimulated TMJ cartilage.

## Data Availability Statement

The raw data supporting the conclusions of this article will be made available by the authors, without undue reservation.

## Ethics Statement

The animal study was reviewed and approved by the Administration Committee of Experimental Animals at the Fourth Military Medical University.

## Author Contributions

YuZ, XX, and PZ contributed to the animal operation, data acquisition and interpretation, draft, and composition of the manuscript. QL, MZ, HY, and YaZ contributed to the data explanation and composition of the manuscript. WH contributed to the animal management and sample preparation. SY and JZ contributed for data acquisition and data interpretation. MW contributed to conception, design, data interpretation, and composition of manuscript. All authors gave final approval and agreed to be accountable for all the aspects of the work.

## Conflict of Interest

The authors declare that the research was conducted in the absence of any commercial or financial relationships that could be construed as a potential conflict of interest.

## Publisher’s Note

All claims expressed in this article are solely those of the authors and do not necessarily represent those of their affiliated organizations, or those of the publisher, the editors and the reviewers. Any product that may be evaluated in this article, or claim that may be made by its manufacturer, is not guaranteed or endorsed by the publisher.
